# Functional Relationships between Lipid Metabolism and Liver Regeneration

**DOI:** 10.1155/2012/549241

**Published:** 2012-01-26

**Authors:** David A. Rudnick, Nicholas O. Davidson

**Affiliations:** ^1^Department of Pediatrics, Washington University School of Medicine, P.O. Box 8208, St. Louis, MO 63110, USA; ^2^Department of Developmental Biology, Washington University School of Medicine, P.O. Box 8208, St. Louis, MO 63110, USA; ^3^Department of Medicine, Washington University School of Medicine, P.O. Box 8208, St. Louis, MO 63110, USA

## Abstract

The regenerative capacity of the liver is well known, and the mechanisms that regulate this process have been extensively studied using experimental model systems including surgical resection and hepatotoxin exposure. The response to primary mitogens has also been used to investigate the regulation of hepatocellular proliferation. Such analyses have identified many specific cytokines and growth factors, intracellular signaling events, and transcription factors that are regulated during and necessary for normal liver regeneration. Nevertheless, the nature and identities of the most proximal events that initiate hepatic regeneration as well as those distal signals that terminate this process remain unknown. Here, we review the data implicating acute alterations in lipid metabolism as important determinants of experimental liver regeneration and propose a novel metabolic model of regeneration based on these data. We also discuss the association between chronic hepatic steatosis and impaired regeneration in animal models and humans and consider important areas for future research.

## 1. Introduction

The liver has remarkable capacity to recover from injury. Such regenerative potential is essential for survival following partial hepatic resection (e.g., for tumor removal or live-donor liver transplantation) and from acute and chronic liver injury secondary to toxins, infections, immune dysfunction, metabolic diseases, or other causes [1–3]. Nevertheless, liver diseases remain an important cause of morbidity and mortality, and inadequate hepatic regeneration likely contributes. Based on these considerations, the mechanisms that regulate liver regeneration continue to be the subject of intense research, with hope that the knowledge gained will lead to novel strategies with which to improve the outcomes of many liver diseases. A number of studies have identified fatty liver as an important risk factor for impaired liver regeneration in humans and experimental animal models. In apparent distinction to those observations however, and still a lingering paradox in the literature, a number of reports suggest that the transient hepatocellular fat accumulation characteristic of early regeneration following partial hepatectomy (PH) in rodents is actually *required* for physiological liver regeneration. Here, we review these data, propose a hypothesis for the seemingly dichotomous relationship of chronic versus acute hepatic fat accumulation on liver regeneration, and consider important areas for future research.

## 2. Experimental Liver Regeneration

The best characterized and most readily controlled experimental model for investigating the molecular, cellular, and physiologic mechanisms that control liver regeneration has been PH in rodents [4]. In the most typically used version of this paradigm, that is, “two-thirds” PH, the anesthetized rodent is subjected to midventral laparotomy with sequential ligation and resection of the left and median hepatic lobes followed by closure of the surgical wounds and recovery. Subsequent regeneration is assessed by analyses of hepatocellular proliferation, liver mass, gene and protein expression, and signaling events at serial time points after the surgery. Studies using this experimental system show that regeneration after PH is precisely regulated in both its initiation and duration, terminating only when the original liver-to-body mass ratio is restored. Furthermore, this response does not require recruitment or mobilization of either a resident or exogenous stem cell population. Rather, all of the normally quiescent hepatocytes in the mature liver have the potential to proliferate in response to partial hepatic resection [5]. Pharmacological and genetic manipulations of animals subjected to PH have identified many signals, including cytokines (e.g., tumor necrosis factor *α* (TNF*α*) and interleukin 6 (IL6)), growth factors (e.g., hepatocyte growth factor (HGF), epidermal growth factor-receptor ligands, and fibroblast growth factors), intracellular signaling events (e.g., Wnt/*β*-catenin), and transcription factors (e.g., NF*κ*B, STAT3, CREB, C/EBP*β*, AP1, FXR, and LXR), that are regulated in response to PH and influence the subsequent hepatic regenerative response (reviewed in [1–3, 5, 6]). Those signals, some of which are initiated within minutes of surgical resection, promote restoration of normal hepatic mass, architecture, and function over the ensuing days, after which regeneration ceases.

One advantage of the PH model of liver regeneration over others is the absence of injury to the remnant liver tissue following the (surgically induced) regenerative stimulus, which thereby minimizes potential confounders to interpretation of the functional specificity of induced signals for the regenerative response itself. Nevertheless, experimental models of toxin-induced liver regeneration (e.g., carbon tetrachloride (CCl_4_), thioacetamide [7, 8]) have also been extensively used to investigate regenerative mechanisms, and the importance of several regulatory pathways identified in the PH model has been shown to be conserved in such paradigms [9, 10]. In contrast, the hepatocellular proliferative response to primary mitogens does not depend upon TNF*α*, HGF, NF*κ*B, and several other signals implicated as important in hepatic insufficiency-induced liver regeneration [11–16]. Despite the broad knowledge gained from almost a century of studies using these models, the nature and identities of the *most proximal events* that initiate hepatic regeneration as well as the distal events that terminate this process are still not known.

## 3. The Metabolic Response to Hepatic Insufficiency and Liver Regeneration

Liver mass is maintained or recovered in precise proportion to body mass, giving rise to the concept of an intrinsic regulator of liver : body mass ratio, that is, the “*hepatostat*” [5, 6]. Amongst its many essential functions, the liver plays critical roles in the regulation of systemic metabolism and extrahepatic energy consumption, which themselves are influenced by body mass [17]. Together, these considerations suggest that changes in intermediary metabolism in response to hepatic insufficiency could contribute essential signals for initiation of liver regeneration, and, conversely, that restoration of metabolic homeostasis after recovery of the normal liver : body mass ratio might provide signals that terminate this response. Several experimental observations support such a metabolic model of liver regeneration.

### 3.1. Glucose Metabolism during Liver Regeneration

Consistent with the liver's central role in gluconeogenesis, rodents subjected to PH become hypoglycemic. Extending this paradigm, other studies show that either intravenous or enteral dextrose supplementation suppresses both PH- [18–24] and toxin- (CCl_4_, thioacetamide; [25, 26] and J. Huang and D.A. Rudnick, unpublished observations) -induced hepatocellular proliferation. Similarly, dietary caloric restriction accelerates onset of hepatocellular proliferation in response to surgical- or toxin-induced hepatic insufficiency [27, 28]. Circulating insulin levels decline in response to PH-induced hypoglycemia (and are augmented by dextrose supplementation [18]), and, interestingly, hepatocellular proliferation in models of toxin-induced liver injury is accelerated and augmented in mice with streptozotocin-induced insulin-deficient diabetes [29, 30]. Nevertheless, it remains to be established if changes in insulin signaling mediate the effects of dextrose supplementation on liver regeneration in such models. Somewhat paradoxically, studies have also shown that insulin supplementation reverses hepatic lobar atrophy in response to portacaval shunting *in vivo* and that insulin augments the activity of hepatocyte mitogens in cell culture [5]. This latter observation is consistent with studies, including those noted above, demonstrating that differences exist between the signals that regulate hepatocellular proliferation in response to surgical- or toxin-induced loss of liver mass and those that determine mitogen-induced proliferation.

Dextrose-mediated inhibition of PH-induced liver regeneration is associated with disruption of many signaling events identified as important for regeneration. For example, provision of supplemental dextrose augments hepatic expression of the mitoinhibitory factors C/EBP*α*, p21, and p27 and suppresses expression of the proregenerative transcriptional regulator, FoxM1, in animals subjected to PH [18]. Consistent with these findings, induction of proregenerative signals, including IL6, transforming growth factor *α* (TGF*α*), and HGF, is accelerated by caloric restriction in toxin- (thioacetamide)-induced liver regeneration [28]. Together, these observations support a model in which the hypoglycemic response to hepatic insufficiency initiates the signals that promote liver regeneration. Many of the regenerative signals that are disrupted by dextrose supplementation are also deranged in association with the impaired regenerative response observed in aged animals subjected to PH [31, 32]. Those changes in older mice appear to be mediated, at least in part, by age-dependent epigenetic effects [33, 34]. Together, these findings support a model in which the metabolic responses to hepatic insufficiency after PH (e.g., hypoglycemia) activate a transcriptional network through pathways including epigenetic regulation. In contrast to PH-induced liver regeneration, toxin-induced regeneration is undiminished in old versus young animals [35]. The mechanisms responsible for this difference are unknown and merit further investigation.

### 3.2. Systemic Catabolism during Liver Regeneration

The observations noted above implicate hypoglycemia and subsequently induced alterations in systemic metabolism as modulators of physiological liver regeneration. This consideration has prompted further investigation of the metabolic response to hepatic insufficiency in experimental models of regeneration. Recent reports have characterized the stereotypical decline in systemic lean and adipose tissue stores and the ensuing rise in circulating and hepatic free fatty acids and specific amino acids that occur in response to PH prior to the onset of regeneration [36–38]. Those analyses also show that specific alterations in metabolism, like the regenerative response itself, occur in proportion to the extent of hepatic insufficiency [36]. For example, two-thirds PH results in a significantly greater loss of systemic adipose stores (and a more robust hepatocellular proliferative response) than does one-third hepatectomy [36]. Systemic fat depletion has also been observed in various models of toxin-induced liver regeneration [36, 39]. These findings together suggest that catabolism of systemic adipose tissue might regulate the hepatic regenerative response to surgical and toxin-induced loss of liver mass.

### 3.3. Hepatic and Systemic Lipid Metabolism during Liver Regeneration

As noted above, it has long been recognized that the early regenerating liver transiently accumulates hepatocellular fat after PH [40–43]. Other work has demonstrated fat accumulation concomitant with cellular proliferation in primary hepatocyte culture [44], raising the possibility that fat accumulation might in turn regulate hepatocyte proliferation. Furthermore, the patterns of hepatic mRNA induction during early PH-stimulated liver regeneration suggest the existence of a conserved transcriptional program leading to regulated transient “steatosis” during the regenerative response [45, 46]. The role of endogenous hepatic lipogenesis in regulating liver regeneration is less clear. Increased *de novo* hepatic fatty acid production has been reported in regenerating liver [43], but mice with liver-specific disruption of fatty acid synthase expression (i.e., FASKOL mice, [47]) exhibit comparable hepatic fat accumulation (and liver regeneration) after PH compared to that in wild-type controls, strongly suggesting that *de novo* hepatic lipogenesis is not required for the development of such transient hepatic steatosis or the regenerative response [37]. These and other findings point to systemic adipose tissue as the primary source of the lipid that accumulates in regenerating liver [39, 40].

## 4. Hepatic Steatosis during Experimental Liver Regeneration

A number of experimental observations provide support for the possibility that the alterations in hepatic and systemic lipid metabolism discussed above are essential for normal liver regeneration. For example, older studies have noted increased dependency following PH of regenerating liver on *β*-oxidation of fatty acids for energy production [22, 48]. Indeed, it has been speculated that the inhibitory effect of dextrose supplementation on liver regeneration might be secondary to the suppressive effect of such supplementation on the release of free fatty acids from systemic adipose stores, and infusion of an inhibitor of *β*-oxidation, ((+)-octanoylcarnitine), has been reported to impair regeneration [48]. Moreover, parenteral administration of lipid emulsions or of carnitine, which mediates uptake of acyl groups into mitochondria for *β*-oxidation, has been reported to accelerate PH-induced regeneration [22], and dietary supplementation with palmitate and carnitine augments toxin- (thioacetamide)-induced hepatocellular proliferation [49]. However, questions about the role of alterations in *β*-oxidation have been raised by analyses of PPAR*α* knockout mice in which *β*-oxidation is dysregulated. Some reports demonstrate normal PH-induced regeneration [37, 50], and others show impaired regeneration [51, 52] in these animals. More recent studies have reported inhibition of liver regeneration by various experimental interventions that decrease hepatic fat accumulation after partial hepatectomy, including both pharmacological (e.g., clofibrate [53], leptin [45], or propranolol [54] supplementation) and genetic (e.g., liver-specific disruption of glucocorticoid receptor expression, [45]) strategies. Those findings collectively imply a requisite role for hepatic steatosis in liver regeneration. However, as alluded to above, other findings raise questions about the specific function of hepatic fat accumulation during normal regeneration. For example, fat accumulation is suppressed but regeneration proceeds normally following PH in liver fatty acid binding protein (L-Fabp) knockout mice [37]. In addition, caveolin 1-null mice exhibit reduced hepatic steatosis after PH, with regeneration reported to be impaired in one study [55] but not another [56]. Finally, regeneration proceeds normally in mice with intestine-specific deletion of the microsomal triglyceride transfer protein (MTP-IKO), which is essential for intestinal absorption of dietary fat and which exhibit decreased peripheral adipose tissue [37]. Importantly, hepatectomy-induced fat accumulation was reduced but not completely abrogated in L-Fabp-null and MTP-IKO mice [37], leading to speculation about the existence of a *threshold of adaptive lipogenesis essential for regeneration* but not influenced in those models. The role of mobilization of lipid from adipose tissue stores during PH- and toxin-induced liver regeneration has also been investigated by analyses of fatty liver dystrophy (*fld*) mice, which have a paucity of systemic adipose tissue as a result of global disruption of *Lpin1* expression [57]. *fld* mice exhibit reduced hepatocellular triglyceride accumulation and proliferation with augmented hepatic p21 expression after PH compared to littermate controls [36]. *fld* mice also display increased mortality in response to CCl_4_-induced liver injury [36]. Taken together, these data support a model in which metabolism of systemic adipose tissue in response to hepatic insufficiency promotes initiation of hepatocellular proliferation. However, they do not establish the mechanisms responsible.

An important caveat to the analyses of *fld* mice is that the target gene of interest, *Lpin1,* is expressed in liver and muscle in addition to adipose tissue, and its expression is globally disrupted in *fld* mice [57]. Thus, *Lpin1* might have effects on hepatic steatosis and hepatocellular proliferation during liver regeneration dependent on its hepatic expression and independent of its effects on systemic adipose tissue stores. Interestingly, hepatic *Lpin1* expression is induced after PH, and such induction is attenuated in liver-specific glucocorticoid receptor null mice, in which (as noted above) the metabolic and hepatocellular proliferative responses to PH are deranged ([45] and D. A. Rudnick, unpublished observations). These findings, together with the known pleiotropic functions of the protein product of *Lpin1* (lipin1, [58]) suggest several potential alternative mechanisms to explain the impaired regenerative phenotype in *fld* mice [36]). For example, lipin1 amplifies peroxisome proliferator-activated receptor gamma coactivator 1*α*- (PGC1*α*-) regulated transcription in hepatocytes to increase expression of genes encoding enzymes involved in fatty acid oxidation (and known to be regulated during liver regeneration, [59, 60]). Lipin1 also stimulates peroxisome proliferator-activated receptor *γ*- (PPAR*γ*-) dependent adipogenic gene expression in adipocytes [61]. Finally, lipin1 is a phosphatidic acid phosphatase enzyme, catalyzing the conversion of phosphatidic acid (PA) to diacylglycerol (DAG, [62]). This reaction plays a key role in triglyceride and phospholipid biosynthesis [63]. PA and DAG also function as lipid second messengers in protein kinase C activation [64], which occurs during liver regeneration [65–67]. Thus, loss of the transcriptional or enzymatic activities of hepatic lipin1 might contribute to impaired regeneration in *fld* mice.

A further consideration, not exclusive of those outlined above, is that alterations in hepatic and circulating pools of cholesterol might play a role in some phases of liver regeneration. This possibility has emerged from analyses in PCSK9 knockout mice, which exhibit decreased pools of circulating cholesterol, impaired regeneration, and hepatic necrosis after PH, all of which are reversed by high cholesterol feeding [68]. Additional support for this idea comes from demonstration that ligand-induced liver X receptor (LXR) activation in mice subjected to PH alters plasma and hepatic cholesterol pools and impairs liver regeneration [69].

## 5. A Metabolic Model of Liver Regeneration

**Figure 1:**
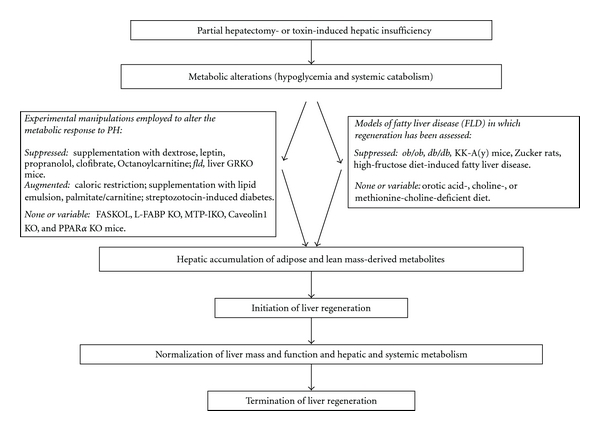
*A metabolic model of liver regeneration*: the data reviewed here implicate the metabolic response to hepatic insufficiency as a source of specific signals that initiate liver regeneration. Experimental manipulations employed to alter this metabolic response that are discussed in the text are listed in the box to the left (along with their reported effects on regeneration: suppressed, augmented, and none or variable effects). Models of fatty liver disease (FLD) in which regeneration has been assessed that are discussed in the text are listed in the box to the right. (*fld*: fatty liver dystrophy mouse; GRKO: glucocorticoid receptor knockout; FASKOL: liver-specific fatty acid synthase knockout mouse; L-Fabp KO: Liver fatty acid binding protein knockout; MTP-IKO: intestine-specific microsomal triglyceride transfer protein knockout; PPAR: peroxisome proliferator-activated receptor).

The data summarized above suggest that alterations in lipid metabolism that occur in response to hepatic insufficiency contribute to the initiation of both resection- and toxin-induced liver regeneration. Although the specific mechanisms that couple changes in lipid metabolism to onset of hepatic regeneration have not been elucidated, several possibilities are worthy of consideration (Figure 1). For example, lipids delivered from the periphery or synthesized *de novo* in response to partial hepatic resection or other liver injury might serve simply as the substrate for energy production [70] or for membrane synthesis required for hepatocellular proliferation. Another intriguing consideration is that lipid-derived metabolites might influence regenerative signaling pathways via transcriptional or epigenetic mechanisms. Several lines of evidence provide indirect support for this latter concept. For example, the transcriptional activities of the nuclear steroid hormone transcription factors PPAR*α*, FXR, and LXR, which have each been implicated as important during PH-induced liver regeneration [51, 52, 69, 71], are regulated by binding to specific classes of phospholipid [72], bile acids [71], or oxysterols [69], respectively. In addition, certain fatty acids have been reported to influence the acetylation state of metabolic enzymes in hepatocytes [73] and thus might also regulate epigenetic changes in regenerating liver. Finally, hepatic insufficiency-induced alterations in lipid metabolism might affect physiologic liver regeneration via adipose-derived hormones. Indeed, the influence of adipokines on regeneration has been suggested by studies showing inhibition of PH-induced liver regeneration in wildtype mice by leptin supplementation [45] and impaired regeneration in adiponectin knockout mice [74, 75].

## 6. Impaired Liver Regeneration in Experimental Models and Humans with Fatty Liver Disease

 The influence of adipose metabolism on liver regeneration has also been demonstrated by the recognized association between chronic hepatic steatosis and impaired regeneration in experimental animal models. Leptin-deficient (*ob/ob*) [76–79] and -resistant (*db/db*) [80, 81], diabetic KK-A(y) [82], “Western” [83] and high-fructose [84] diet-fed mice, and leptin-resistant obese Zucker rats [85, 86], each of which exhibit hepatic steatosis, have all been reported to demonstrate impeded regeneration after PH or CCl_4_ administration. In contrast, liver regeneration is not impaired in models of mild hepatic steatosis, including orotic acid- [86] and choline- [87] deficient diet-fed rats, leading some investigators to speculate that the degree of steatosis is important in determining its effect on liver regeneration. Consistent with that interpretation, liver regeneration is variably affected in animals administered a methionine-choline deficient (MCD), a phenotype dependent on the magnitude of steatosis [86, 88–91]. Despite these many studies linking chronic hepatic steatosis with impaired liver regeneration, the mechanisms responsible remain enigmatic. Moreover, the basis for the differences in the influence of chronic and acute hepatic fat accumulation on regeneration is undefined.

Chronic steatosis has also been associated with adverse outcomes after major hepatic resection in humans. A recent meta-analysis showed that the risk of postoperative complications in patients with any degree of steatosis undergoing hepatectomy (for neoplasm) was double that of their nonsteatotic counterparts, and that those with excessive (>30%) steatosis had an almost 3-fold increased risk of death [92]. This analysis did not address whether impaired regeneration was the culprit; however, a study of patients undergoing liver resection (for living related liver donation) showed reduced recovery of liver volume over the initial 3 months following surgery in patients with mild steatosis (versus no steatosis, [93]), and another study reported decreased recovery of liver function 6–12 months after hepatectomy in such patients [94]. These findings are consistent with the animal model studies discussed above.

## 7. Summary and Future Investigations

As enumerated above, extensive older and more recent analyses implicate alterations in adipose metabolism in response to surgical- or toxin-induced hepatic insufficiency as functionally important for initiation of normal liver regeneration. However, the molecular basis for these effects has not yet been elucidated. Similarly, the mechanisms responsible for the inhibitory effect of chronic hepatic steatosis on liver regeneration in experimental model systems and humans undergoing hepatic resection remain to be established. It is tempting to speculate that the acute changes in systemic lipid metabolism that occur in response to hepatic insufficiency have specific, direct transcriptional, and epigenetic proregenerative effects, and that such events are modified or reversed in chronic fatty liver disease. Future studies should investigate the functional relationships between these metabolic, genetic, and epigenetic alterations during normal liver regeneration and examine the influence of chronic hepatic steatosis on those relationships.

## Abbreviations

CCl_4_: Carbon tetrachloride
DAG: Diacyl glycerol
FLD: Fatty liver disease
*fld*: Fatty liver dystrophy mouse
FASKOL: Liver-specific fatty acid synthase knockout
GRKO: Glucocorticoid receptor knockout
HGF: Hepatocyte growth factor
IL6: Interleukin 6
L-Fabp KO: Liver fatty acid binding protein knockout
LXR: Liver X receptor
MTP-IKO: Intestine-specific microsomal triglyceride transfer protein knockout
PH: Partial hepatectomy
PA: Phosphatidic acid
PGC1*α*: Peroxisome proliferator-activated receptor *γ* coactivator 1*α*
PPAR: Peroxisome proliferator-activated receptor
TGF: Transforming growth factor
TNF*α*: Tumor necrosis factor *α*.
